# Validity and reliability of the Amharic version Revised Child Anxiety and Depression Scale (RCADS-25) in pediatric oncology

**DOI:** 10.1186/s40359-026-04922-7

**Published:** 2026-06-09

**Authors:** Tenaw Gualu Melesse, Janita Pak Chun Chau, William Ho Cheung Li, Kai Chow Choi, Mulugeta Ayalew Yimer, Abdulkadir Mohamedsaid Gidey

**Affiliations:** 1 The Nethersole School of Nursing, Faculty of Medicine, The Chinese University of Hong Kong, Hong Kong, HKSAR China; 2 Department of Pediatrics and Child Health Nursing, College of Medicine and Health Sciences, Debre Markos University, Debre Markos, Ethiopia; 3 Department of Rehabilitation Sciences, Faculty of Health and Social Sciences, The Hong Kong Polytechnic University, Hong Kong, HKSAR China; 4 Pediatric Hematology-Oncology Unit, Department of Pediatrics and Child Health, School of Medicine, University of Gondar, Gondar, Ethiopia; 5 Pediatric Hematology-Oncology Division, Department of Pediatrics and Child Health, Faculty of Medicine, Addis Ababa University, Addis Ababa, Ethiopia

**Keywords:** Cancer, Child, Oncology, Reliability, Revised child anxiety and depression scale, Validity

## Abstract

**Background:**

The short form of the Revised Child Anxiety and Depression Scale (RCADS-25) has been frequently used for assessing anxiety and depression in clinical and community samples. However, the Amharic version of RCADS-25 (RCADS-25(A)) has not been validated. This study aimed to evaluate the validity and reliability of RCADS-25(A) in pediatric oncology.

**Methods:**

This study included 142 children aged 8–18 years. The reliability of the scale was assessed by calculating internal consistency and test-retest reliability. Convergent validity was assessed by examining the correlation between the RCADS-25(A) and the Pediatric Quality of Life Inventory 4.0 Generic Core Scale (PedsQL™ 4.0 GCS). Confirmatory factor analysis was conducted to evaluate the two-factor structure of RCADS-25 identified in the original version.

**Results:**

The mean age of the participants was 11.6 ± 3.2 years. The RCADS-25(A) demonstrated evidence of good validity and reliability. Cronbach’s alpha coefficients were 0.95, 0.94, and 0.96 for the anxiety subscale, depression subscale, and total scale, respectively, while intraclass correlation coefficients were 0.81, 0.80, and 0.87 for the same domains, indicating good test–retest reliability. The RCADS-25(A) correlated significantly with PedsQL™ 4.0 GCS (*r* = -0.97, *p* < 0.001). Confirmatory factor analysis supported the two-factor model (χ2/df = 1.44; CFI = 0.95; RMSEA = 0.06; SRMR = 0.05; TLI = 0.95).

**Conclusions:**

The RCADS-25(A) appears to demonstrate promising validity and reliability, suggesting its potential utility for assessing anxiety and depression in pediatric oncology. The findings underscore the need for further validation studies employing larger and more diverse samples, as well as the adoption of multimethod assessment approaches. The use of parent-report versions and studies in other populations and Ethiopian languages are highlighted to further establish the scale’s psychometric properties and cross-cultural applicability.

## Background

Childhood cancer was the sixth and ninth leading cause of the total cancer burden and childhood disease burden worldwide in 2017, respectively [1]. In Ethiopia, childhood cancer incidence rates have been increasing over time [2], and more than 6,000 new cases were estimated in 2020 [3]. Children with cancer often report substantial emotional distress throughout their illness trajectory. One study reported the prevalence of emotional and/or behavioral disorders in nearly 90% of pediatric cancer patients [4], of which anxiety and depression are most frequently reported [5, 6].

Anxiety and depression significantly impact the quality of life, disease prognosis [7, 8], functional status [9], treatment adherence [10] and increase off-therapy distress symptoms [11]. Thus, an age-appropriate systematic assessment of anxiety and depression using appropriate instruments can help identify patients’ needs and initiate effective interventions.

The Revised Child Anxiety and Depression Scale (RCADS) is one of the most frequently used measures of anxiety and depression symptoms in children and adolescents aged 8–18 years old [12]. Originally, it was a 47-item scale developed by adapting Spence’s children’s anxiety measure in correspondence with the Diagnostic and Statistical Manual of Mental Disorders (DSM-IV) [13]. Later, short forms of the RCADS were validated in clinical and community samples, from which a 25-item self-report version of the RCADS was frequently used. The RCADS-25 contains two subscales: a 15-item broad anxiety subscale and a 10-item depression subscale. The RCADS-25 has demonstrated satisfactory psychometric properties in the original version among clinical samples (Cronbach’s alpha (α) = 0.91 and 0.80 for the anxiety and depression subscales, respectively) [14], and has been translated and validated in different languages for cross-cultural research.

The RCADS has advantages over most other self-reported anxiety and depression measures, as it is available free of charge and contains both anxiety and depression subscales that can be administered together. Moreover, the short version of RCADS (RCADS-25) is more feasible in a clinical setting because it reduces the time required to administer the scale and thus eases the burden for participants, particularly young children [14].

Although children with cancer often experience emotional distress, to date, there is limited published evidence on the magnitude of anxiety and depression in pediatric oncology in Ethiopia. Additionally, no appropriate instruments to assess anxiety and depression symptoms are available in Amharic, which is the most widely spoken and written language in Ethiopia [15]. Thus, this study aimed to evaluate the validity and reliability of the Amharic version of RCADS-25 for Ethiopian children with cancer.

## Methods

### Study design

This study was part of a larger psychometric evaluation study; detailed methods have also been reported elsewhere [16]. The study included two phases. First, the original English version of RCADS-25 [14] was translated into Amharic and evaluated for content validity. Then, the psychometric properties of the Amharic version of RCADS-25 (RCADS-25 (A)), including internal consistencies, test-retest reliability, convergent validity, and factorial validity, were examined using a descriptive cross-sectional study.

### Translation and content validation

First, we obtained permission from the original author to translate the RCADS-25 to Amharic. The translation was carried out according to the model of back translation [17], consisting of forward-translation, review of the translated version, back-translation, and review of translation equivalence [17]. The English version of the RCADS-25 [14] was translated into Amharic by a bilingual pediatric nurse who was familiar with the subject matter and had experience with pediatric cancer patients. Then, an Amharic teacher was invited to review the Amharic version of the RCADS-25. The reviewer identified any difficulties in wording and comprehensibility and revised it to make it clear for native Amharic speakers. A second bilingual translator with a pediatric nursing background then blindly back-translated the Amharic version of RCADS-25 to English. The bilingual translators discussed and revised it accordingly whenever there was a discrepancy between the two versions.

A panel of six bilingual experts, including a nurse researcher, a pediatric oncologist, an oncology nurse, a psychiatric nurse, a psychologist, a pediatrician, and a pediatric nurse, evaluated the appropriateness of the translation and the content relevance of each item to anxiety and depression for children and the cultural context. To determine the content validity of the RCADS-25 (A), each was evaluated on a four-point Likert scale (1 = not relevant, 2 = somewhat relevant, 3 = quite relevant, and 4 = highly relevant) [18]. The item-level content validity index (I-CVI) and scale-level content validity index (S-CVI) were determined by the proportion of experts who rated the items 3 or 4 and the average of I-CVI for all items, respectively [19].

A cognitive debriefing was conducted with 10 randomly recruited participants from one of the study hospitals to assess the relevance and comprehensibility of the scale items in relation to participants’ experiences, health status, and ease of scale use. Cognitive debriefing facilitates the evaluation of language clarity and interpretability, developmental appropriateness, conceptual ambiguity, and the time required to complete the scale [20, 21]. Participants’ feedback was reviewed and the scale was revised accordingly.

### Psychometric evaluation of RCADS-25 (A)

#### Study setting and sample

The study participants were recruited from pediatric hematology/oncology outpatient and inpatient departments in four specialized hospitals in Ethiopia from January to April 2022. Children aged eight to 18 years with any type of cancer at any stage of the cancer trajectory, able to communicate in Amharic, and able to provide oral assent and written parent consent were included in the study. Children unable to complete the scale due to an illness were excluded from the study.

The sample size was calculated using a minimum requirement of a 5:1 subject-to-item ratio [22]. Expecting a 10% non-response rate, we targeted to recruit a total of 139 participants consecutively.

### Measurements

The questionnaires comprise sociodemographic and clinical data sheets, RCADS-25 (A), and Paediatric Quality of Life Inventory 4.0 Generic Core Scale (PedsQL™ 4.0 GCS (A)).

The RCADS-25 (A) comprises two subscales: anxiety (15 items) and depression (10 items). Each item was rated on a four-point Likert scale (0 = never, 1 = sometimes, 2 = often and 3 = always) [12]. The total score for each subscale was computed by the sum of the scores of the items that comprise each subscale. The total raw scores were converted into T-scores, with values of 65–70 indicating a borderline clinical range and T-scores of ≥ 70 indicating the clinical range [23]. The scale has shown strong reliability and validity (Cronbach’s α = 0.91 for the total anxiety scale and Cronbach’s α = 0.80 for the depression scale) among clinical samples in the original version [14], and has been translated and validated in several languages for cross-cultural use.

The correlation between RCADS-25 (A) and PedsQL™ 4.0 GCS (A) was used to determine the convergent validity of the RCADS-25 (A). PedsQL™ 4.0 GCS (A) was used to measure the quality of life [24]. The scale contains 23 items and four subscales, including physical functioning (8 items), emotional functioning (5 items), social functioning (5 items), and school functioning (5 items). Each item was scored on a five-point Likert scale (0 = never, 1 = almost never, 2 = sometimes, 3 = often, and 4 = almost always). The total score was calculated by reversing the scoring of the 0–4 scale items and linear transformation to 0–100 (0 = 100, 1 = 75, 2 = 50, 3 = 25, 4 = 0). A higher score refers to a higher quality of life [25]. The scale had strong validity and high reliability in the original study (α = 0.88) [24]. We concurrently conducted a psychometric evaluation for PedsQL™ 4.0 GCS (A) and the scale had strong validity and reliability (Cronbach’s α = 0.96). We obtained permission to use the PedsQLTM 4.0 GCS (A) from Mapi Research Trust on behalf of the author.

### Data collection

Research assistants at each hospital approached participants during their medical appointments to determine their eligibility and interest in participating in the study. After written parental consent, the research assistants collected the clinical data from the patient’s medical records and invited the participants to complete a sociodemographic questionnaire, the RCADS-25 (A), and PedsQL™ 4.0 GCS (A). For the assessment of test-retest reliability of the RCADS-25 (A), the scale was re-administered to 50 participants (mean age = 11.9 years, SD = 2.9) two weeks after the initial administration [26]. Participants for the retest were randomly selected from the overall sample and received a telephone reminder one day before the second administration to minimise attrition.

### Analysis

The data were analyzed using IBM SPSS Statistics 23. Normality for the continuous data was checked through the kurtosis and skewness tests. Demographic and clinical data were summarized using descriptive statistics, including frequency, percentage, mean and standard deviations. The scores for RCADS-25 (A) and PedsQL™ 4.0 GCS (A) were summarized using mean and standard deviation.

The content validity of the RCADS-25 (A) was calculated via content validity index (CVI). The S-CVI score of 0.9 and an I-CVI score of 0.78 were considered acceptable [19].

The internal consistency of the total RCADS-25 (A) and its subscales was assessed by calculating Cronbach’s α, corrected item-to-item correlations, and average inter-item correlations. Cronbach’s α values > 0.9 were considered excellent [27]. Additionally, average inter-item correlations within a range of 0.15–0.5 [28] and corrected item-total correlations of > 0.3 [29], were considered acceptable. The test-retest reliability was examined by computing the intraclass correlation coefficient (ICC) using a single-measurement absolute agreement and a 2-way mixed-effects model, with a value of ≥ 0.75 indicating good reliability [30].

Convergent validity was assessed using the correlation between RCADS-25 (A) and PedsQL™ 4.0 GCS (A). We hypothesized that quality of life has a strong negative correlation with depression [31] and a weak to moderate negative correlation with anxiety [32]. Pearson correlation coefficients of 0.70–0.89 and ≥ 0.9 were considered strong and very strong correlations, respectively [33].

The factorial validity was assessed by using confirmatory factor analysis (CFA). CFA was performed using IBM SPSS Analysis of Moment Structures (AMOS) version 23 to test the fit of the two-factor structure of RCADS-25 reported in the original version [14]. The parameters were estimated using the maximum likelihood estimation method with bootstrapping. First, the multivariate outliers were checked using Mahalanobis distance at *p* < 0.001, and a multivariate normal distribution was considered when the absolute value of skewness was between − 3 and + 3, and kurtosis of -10 to + 10 [34]. The acceptable model fit was evaluated by different indices, including χ2 statistics to the degree of freedom ratio (χ2/df ≤ 3), comparative fit index (CFI) ≥ 0.95, Nonnormed Fit Index (NNFI) also known as Tucker–Lewis index (TLI) ≥ 0.95, root mean square error of approximation (RMSEA) ≤ 0.08, and standardized root mean square residual (SRMR) ≤ 0.1 [35].

## Results

### Content validity of RCADS-25 (A)

The results show that the I-CVI ranged from 0.83 to 1.0 and the S-CVI was 0. 93. Provided that both content validity indexes were above the minimum recommended levels, the RCADS-25 (A) demonstrated good content validity. No revision or deletion of the items was recommended. Moreover, all 10 participants reported no difficulties in interpreting the items or any developmental challenges in completing the RCADS-25 (A). Participants required 15–20 min to complete the demographic data sheet and both scales, which is noteworthy given that the RCADS-25 (A) alone takes an average of 5–10 min to complete.

### Psychometric evaluation of RCADS-25 (A)

#### Participant characteristics

The study involved a total of 142 participants. The participants’ age ranged from 8 to 18 years, with a mean age of 11.6 years (SD, 3.2). Most participants were primary schoolers (84.5%), rural residents (69.7%) and Christians (87.3%). Nearly two-thirds of them were diagnosed with hematological malignancies, and most of the participants (78.2%) were treated with chemotherapy (Table 1). As this is a part of a larger study, the characteristics of the participants were reported in another publication [16].

**Table 1:** Characteristics of the participants (*N* = 142)

Participant characteristics		*n* (%)
Mean age (years) = 11.6 (3.2)		
Gender	Male	82 (57.7)
	Female	60 (42.3)
Education	Grades 3−8	120 (84.5)
	Grades 9−12	22 (15.5)
Religious affiliation	Christian	124 (87.3)
	Muslim	18 (12.7)
Residential address	Rural	99 (69.7)
	Town	43 (30.3)
Family monthly income (Ethiopian birr/USD)	< 2500 (< 48.1)	48(33.8)
	≥ 2500−5000 (≥ 48.1–96.2)	61 (43.0)
	≥ 5000–10,000 (≥ 96.2-192.3)	26(18.3)
	≥ 10,000(> 192.3)	7(4.9)
Cancer diagnosis	Hematological malignancies	94 (66.2)
	Wilms’ tumor	13 (9.2)
	Rhabdomyosarcoma (RMS)	9 (6.3)
	Retinoblastoma	8 (5.6)
	Osteosarcoma	7 (4.9)
	Ewing Sarcoma	6 (4.2)
	Others*	5 (3.5)
Time since diagnosis	< 6 months	78(54.9)
	≥ 6−12 months	34 (23.9)
	≥ 12 months	30 (21.1)
Chemotherapy status	Not started	9 (6.3)
	On treatment	128 (90.1)
	Completed	5 (3.5)
Treatment regimens	Chemotherapy alone	110 (82.7)
	Combination treatment	23 (17.3)
Duration on treatment	< 6 months	76 (57.1)
	≥ 6−12 months	30 (22.6)
	≥ 12−60 months	27 (20.3)

*Neuroblastoma, skin cancer and soft tissue sarcoma; *USD* United States Dollar; 1 USD ~ 52 Ethiopian birr (during data collection)

### Reliability

The Cronbach’s α for the anxiety subscale, depression subscale, and the total RCADS-25 (A) was 0.95, 0.94, and 0.96, respectively, indicating that the subscales and total RCADS-25 (A) have excellent internal consistency. The mean corrected item-total correlations for the anxiety subscale, depression subscale, and total scale were 0.74 (range 0.68–0.79), 0.74 (range 0.70–0.79), and 0.71 (range 0.61–0.78), respectively, in line with the recommended value of above 0.30 [29]. The average inter-item correlations were 0.58 for the anxiety subscale, 0.59 for the depression scale, and 0.51 for the total scale. Likewise, the average inter-item correlations for individual items in the anxiety subscale, depression subscale, and total scale ranged from 0.54 to 0.61, 0.56–0.62, and 0.44–0.58, respectively, indicating a potential risk of item redundancy. However, the removal of items with relatively higher inter-item correlation resulted in only marginal reductions in the Cronbach’s α values for the subscales and total scale, indicating that these items positively contribute to the reliability of the scale. Following a thorough review by expert panels to assess potential item redundancy, the items were determined to be conceptually distinct and were therefore retained in the final scale.

The ICC for the anxiety subscale, depression subscale and the total RCADS-25 (A) was 0.81 (95%CI, 0.69–0.89), 0.80 (95%CI, 0.67–0.88) and 0.87 (95%CI, 0.78–0.92), respectively, indicating good test-retest reliability for both the subscales and the total scale. There was no participant attrition during the test–retest reliability assessment, and no known clinical changes were reported over the two-week interval.

### Convergent validity

The RCADS-25(A) and its subscales were significantly correlated with the PedsQL™ 4.0 GCS (A) and its subscales. The correlation between the total scores of the RCADS-25 (A) and PedsQL™ 4.0 GCS (A) was statistically significant with a very strong negative correlation (*r* = -0.97) (Table 2). However, this exceptionally strong negative association between the theoretically related yet distinct scales should be interpreted with caution and warrants further investigations in future studies.

**Table 2:** The correlations between RCADS-25(A) and PedsQL™ 4.0 GCS (A) (*N* = 142)

	Mean (SD)	Correlation with PedsQL™ 4.0 GCS total score and its sub-scales				
RCADS-25		Health and activities	Feelings	Get along with others	About school	Total PedsQL™ 4.0 GCS
Anxiety subscale	50.68 (11.3)	-0.78^*^	-0.88*	-0.79*	-0.77*	-0.92*
Depression subscale	51.25 (11.2)	-0.77*	-0.79*	-0.71*	-0.69*	-0.86*
RCADS-25 total score	51.04 (11.5)	-0.84*	-0.90*	-0.82*	-0.80*	-0.97*

*p < 0.001

### Factorial validity

**Fig. 1:**
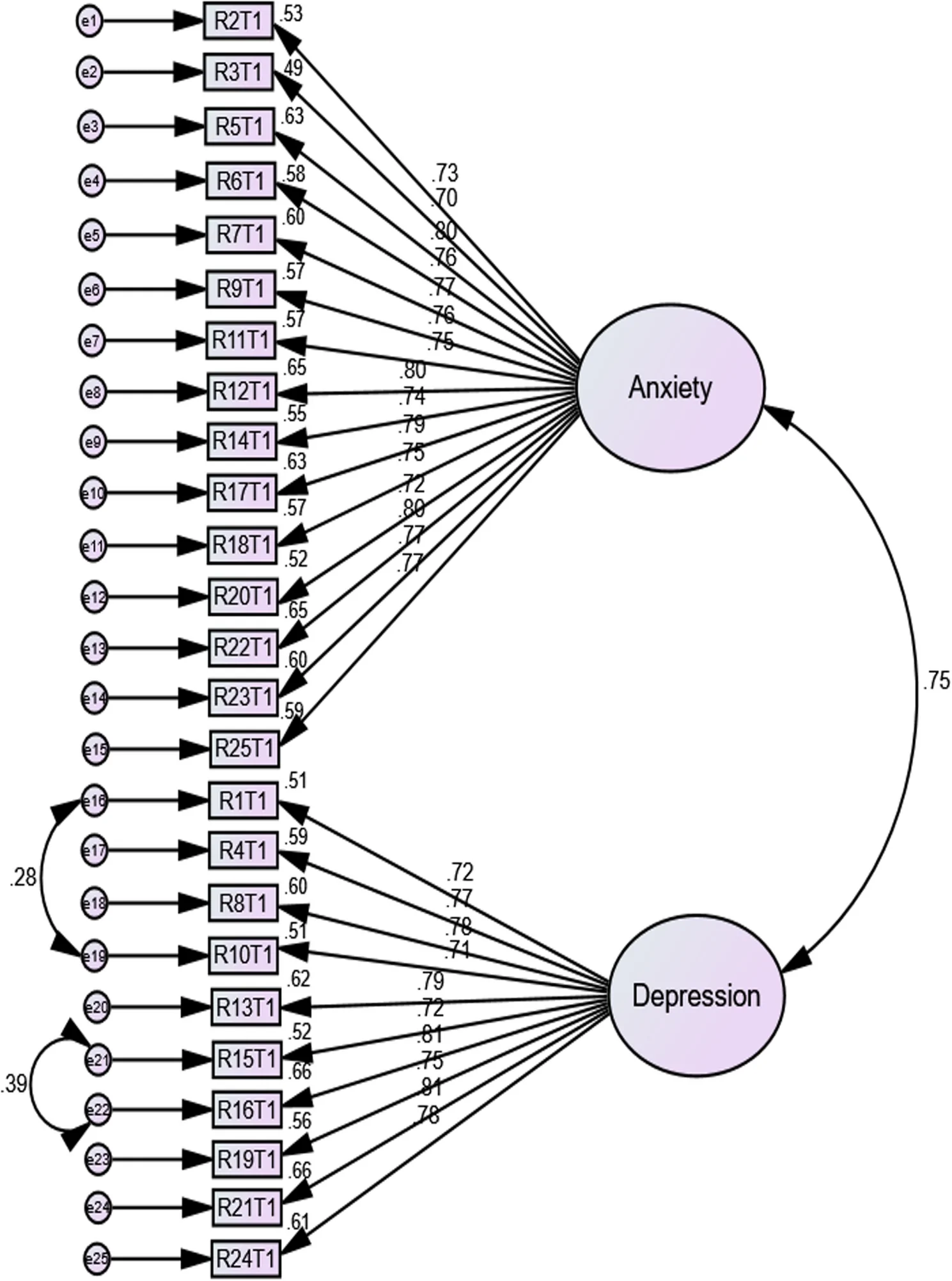
Confirmatory factor analysis model for RCADS-25(A)

In the CFA, we evaluated the broad two-factor structure of the RCADS-25 reported in the original validation study [14]. The factor loadings ranged from 0.70 to 0.81 and were in the acceptable range (Fig. 1). As the maximum likelihood method of estimation assumes multivariate normal distribution, the multivariate outliers and normal distribution were checked. Although no multivariate outliers were detected using the Mahalanobis distance at *p* < 0.001 [34], the joint multivariate kurtosis was 42.45, with a critical ratio of 6.88. Thus, to address multivariate non-normality and improve model fit, the Bollen-Stine bootstrap test with 10,000 bootstrapped samples was conducted [36], and covariance paths were added guided by large modification index values and expected parameter changes [37]. Using a conservative modification index value of ≥ 10, two residual covariances were freed between the error terms e16 and e19, and between the error terms e21 and e22. The original model estimate showed that freeing the covariances between e21 and e22, and between e16 and e19, would reduce the chi-square values by at least 18.86 and 11.40, respectively, and increase the parameter estimates by 0.07 and 0.06, respectively. Additionally, the variables were part of the same subscale, and the positive parameter changes suggest a positive correlation, making the association between these variables theoretically plausible. The bootstrapped test revealed a non-significant result (*p* = 0.09), indicating the stability of the model with non-normal data. The correlation between the anxiety and depression latent factors was 0.75. The modified model fit was evaluated by different indices, and the results supported the two-factor model (χ2/df = 1.44; CFI = 0.95; RMSEA = 0.06; SRMR = 0.05; TLI = 0.95) (Fig. 1). Although maximum likelihood estimation with bootstrapping provides a practical compromise for ordinal data, it assumes multivariate normality, and applying it to a four-point Likert scale may result in slightly biased parameter estimates. In addition, this study focused on testing the two-factor structure identified in the original RCADS-25 [14], and therefore, alternative structural models (e.g., one-factor or bifactor models) were not examined. Therefore, the results should be interpreted in light of these limitations, and future studies employing alternative estimation methods, such as robust maximum likelihood, and comparing alternative models are warranted to further validate these results.

## Discussion

This study aimed to translate and validate the RCADS-25 (A) in Ethiopian children with cancer. To the best of our knowledge, this study is the first to translate the RCADS-25 to Amharic and evaluate its psychometric properties. The RCADS-25 demonstrates promising psychometric properties in this study, exhibiting good validity and reliability. The item-level and scale-level content validity of the RCADS-25 (A) could support the relevance of each item to anxiety and depression in children, as well as the scale’s cultural relevance in the study samples and setting.

The RCADS-25 (A) demonstrated acceptable Cronbach’s α for both the total scale and its subscales. The values were higher than the recommended value of > 0.7 [27] and are congruent with previous studies, including the original version [14, 38]. All corrected item-total correlations for the subscales and total scale were positive and met the recommended threshold (> 0.3) [29]. Although the average inter-item correlations for all items and each item in the scales were slightly above the acceptable range [28], the removal of these items led to a reduction in the Cronbach’s α. Furthermore, detailed item evaluation did not indicate redundancy, and thus no item was excluded from the original scale. These findings suggest that the scale demonstrates satisfactory reliability within the current study context and population.

The RCADS-25 (A) also had good ICC values for the total scale, anxiety subscale, and depression subscale. The ICC values were above the recommended value of ≥ 0.75 [30], and were in line with previous studies [14, 38]. The findings may demonstrate the scale’s stability as shown by consistent results across repeated measurements [39].

The strong correlations between RCADS-25 (A) and PedsQL™ 4.0 GCS (A), as well as their respective subscales, may provide evidence in support of the convergent validity of RCADS-25(A). The findings were consistent with previous literature that revealed anxiety and depression have a weak to strong negative correlation with quality of life [31, 32]. Stronger convergent validity reflects an instrument’s capability to measure constructs that are theoretically related to those assessed by another measure [40]. However, this unusually strong association between theoretically related but distinct scales might arise from methodological issues, including conceptual overlap, scoring artifacts, and shared method variance, rather than a true representation of the relationship between constructs. First, the RCADS-25 (A) and the PedsQLTM 4.0 GCS (A) partly assess similar experiences and show conceptual overlap, particularly within the emotional and physical domains. Second, the scales are scored in opposite directions, and employ different Likert response formats, which may contribute to the magnitude of the negative correlation. Third, as both scales were administered simultaneously using the same self-report Likert format, there is a high likelihood of shared response tendencies, which may substantially inflate the observed correlations rather than the true near-opposite relationship. Accordingly, the findings should be interpreted with caution as the scales measure distinct outcomes that are not directly related and require future studies using diverse samples and multimethod assessments.

The two-factor structure of the RCADS-25 (A) in the current study is consistent with that reported in the original validation study [14], suggesting that the scale retains its intended factorial structure in this population and may adequately capture the targeted constructs among Ethiopian pediatric cancer patients.

Overall, the findings suggest that the RCADS-25 (A) is a promising psychometric measure for anxiety and depression, both in clinical assessment and research among Ethiopian children with cancer. The measurement qualities may support its use in early identification and intervention efforts targeting childhood anxiety and depression in pediatric oncology.

### Limitations of the study

The findings of this study should be interpreted in light of several limitations. First, as the study involved only Amharic-speaking Ethiopian pediatric cancer patients, the generalizability of the findings to other populations may be limited. Second, the relatively moderate sample size suggests that the results should be regarded as preliminary rather than conclusive. Third, in the absence of a publicly available and validated instrument for assessing anxiety and depression in Ethiopian pediatric cancer patients, criterion validity could not be established against a standard measurement. Lastly, we did not assess the caregiver-report version of the RCADS-25 (A) or the disease-specific PedsQL™ 3.0 Cancer Module, nor did we compare these with the child’s self-report version of the RCADS-25 to determine the most appropriate measures. This was because children with chronic illnesses, such as those with cancer who are at high risk of fatigue or have limited literacy, may find it difficult to complete multiple scales simultaneously.

### Implications of the study

The observed evidence of reliability and validity of the RCADS-25 (A) may indicate the scale captures the intended constructs and could be considered culturally appropriate for use among Ethiopian children with cancer. Thus, the RCADS-25 (A) may be used as a rapid anxiety and depression assessment tool in pediatric oncology. Given that the RCADS-25 is to be freely available, contains both anxiety and depression subscales, and has a relatively short test administration time, which can reduce the burden on participants, it may be used in a clinical setting, including for younger children.

Given the study’s moderate sample size, the findings should be considered preliminary and warrant further validation through studies employing larger and more diverse samples, as well as multimethod assessment approaches. As Ethiopia has rich linguistic and cultural diversity [41] and many children communicate only in their mother tongue, further validation studies for non-Amharic-speaking children are warranted. Studies that validate parent-report RCADS-25(A) and compare it with child-report RCADS-25(A) are highlighted to identify the more appropriate scale to assess anxiety and depression in the study population.

## Conclusions

The RCADS-25(A) appears to demonstrate promising validity and reliability, suggesting its potential utility for assessing anxiety and depression in pediatric oncology in both research and clinical contexts. The findings highlight the need for further validation through studies involving larger and more diverse samples, as well as the application of multimethod assessment approaches. In addition, future research could benefit from incorporating parent-report versions and extending validation to other populations and Ethiopian language contexts to more comprehensively evaluate the scale’s psychometric properties and cross-cultural applicability.

## Data Availability

The data supporting the findings of this study can be obtained from the primary author upon reasonable request.

## Abbreviations

CFA: Confirmatory factor analysis
CFI: Comparative fit index
CI: Confidence interval
DF: Degree of freedom
ICC: Intraclass correlation coefficient
I-CVI: Item content validity index
PedsQL™ 4.0 GCS: Pediatric Quality of Life Inventory 4.0 Generic Core Scale
RCADS-25: 25-item Revised Child Anxiety and Depression Scale
RCADS-25(A): Amharic version 25-item Revised Child Anxiety and Depression Scale
RMSEA: Root mean square error of approximation
S-CVI: Scale content validity index
SRMR: Standardized root mean square residual
TLI: Tucker–Lewis index
χ2: Chi square statistics
